# “How do we want to be led?” Team aspirations and adaptive leadership in virtual and hybrid work

**DOI:** 10.1080/17482631.2026.2673289

**Published:** 2026-05-12

**Authors:** Cass Coulston, Sukhi Shergill, Ricardo Twumasi, Myanna Duncan

**Affiliations:** a King’s College, London. Institute of Psychiatry, Psychology and Neuroscience, London, United Kingdom; b Kent and Medway Medical School, Canterbury, UK

**Keywords:** Virtual teams, hybrid teams, team well-being, job demands-resources, leadership dynamics

## Abstract

**Purpose:**

As virtual and hybrid work become embedded in organisational life,understanding how leadership can best support well-being and effective team functioning is vital. This study explores how leadership is experienced and enacted in virtual and hybrid teams, using the Job Demands–Resources (JD-R) model as an interpretive framework and Conservation of Resources (COR) theory to deepen interpretation of strain and sustainability over time.

**Methods:**

Qualitative data were gathered from thirty interviews with leaders and twenty-nine focus groups with team members (*n*  =  110) across multiple industries and geographies. Data were analysed using reflexive thematic analysis.

**Results:**

Three overarching themes were identified: “Connected Leadership Aspirations,” “Leadership as a Positive Social Influence,” and “The Leadership Tension.” Findings highlighted a strong convergence between leaders’ and team members’ aspirations for relational, emotionally present leadership, while also revealing the strain leaders experienced in sustaining this form of leadership amid low visibility, emotional labour, and limited systemic support.

**Conclusion:**

This study extends understanding of leadership in virtual and hybrid teams by showing how leadership can be both supportive and demanding. Interpreted through JD-R and COR, the findings highlight the need for organisational systems and development approaches that adequately resource leadership demands to support well-being, connection, effective team functioning, and leadership sustainability.

## Introduction

As organisations become increasingly globalised and digitised, virtual and hybrid working arrangements have transformed how teams operate and how leadership is enacted (CIPD, 2022; Raghuram et al., 2019). Hybrid teams, which coordinate work across both digital and in-person settings, differ markedly from traditionally co-located teams in how they communicate, make decisions, and sustain cohesion (Balbinot et al., 2024; Hincapie & Costa, 2024). In such contexts, where physical presence is inconsistent or limited, leadership relies less on visibility and positional authority and more on relational presence, trust and social influence (Castellano et al., 2021; Coulston et al., 2025a).

Since the COVID-19 pandemic, a growing body of research has highlighted how virtual and hybrid work reshapes not only coordination and performance, but also the social and psychological conditions of work. Post-pandemic studies highlight heightened risks of isolation, reduced informal support, digital fatigue, and uneven access to information and influence for team members working across dispersed settings (Balbinot et al., 2024; Coulston et al., 2025a). In response, leadership expectations have shifted away from command-and-control models toward more relational and adaptive approaches that prioritise trust, emotional support, and intentional connection and empowerment (Badrinarayanan, 2024; Coulston et al., 2025b; Efimov et al., 2022). These shifts reflect a broader post-pandemic recalibration of leadership expectations, where team members increasingly value connection, authenticity - understood here as leaders being perceived as genuine and consistent - and adaptability from their leaders (Badrinarayanan, 2024; Castellano et al., 2021). Leadership in hybrid contexts is therefore increasingly understood as an emergent and relational process, shaped through distributed influence, and collective sense-making rather than formal authority alone (Lin et al., 2019; Torre & Sarti, 2020).

Coupled with these shifts, scholars have drawn attention to the implications of virtual and hybrid work for well-being. Research on emotional labour, defined here as the regulation and management of emotions and emotional responses in line with role and relational expectations, has accelerated in recent years, extending beyond frontline service roles to encompass leadership and collaboration in knowledge-based and digitally mediated work (Gabriel et al., 2023). This highlights how sustained emotional regulation, availability, and impression management can contribute to strain and depletion over time, particularly in contexts characterised by uncertainty and limited feedback.

Despite the growing prevalence of virtual and hybrid work, systematic reviews indicate that the implications of leadership and well-being in virtual and hybrid teams remain under-theorised and methodologically fragmented (Banker et al., 2023; Coulston et al., 2025b; Tummers & Bakker, 2021). While leadership continues to be a well-researched field, much of the existing research remains focused on established leadership styles such as transformational leadership, often examined through predefined constructs or self-report surveys. While such models offer valuable insight, they do not fully capture how leadership emerges in-the-moment or evolves through relational interactions, particularly under conditions of reduced visibility, distributed work, and changing team expectations.

Emotional and relational dimensions of leadership are also underexplored, especially in relation to how they are experienced by both leaders and their teams across different virtual and hybrid configurations (Hincapie & Costa, 2024). Leadership is often assumed to function as a resource that supports effective team functioning, well-being and culture. In virtual and hybrid contexts, leadership may also operate as a source of demand, requiring leaders to navigate dispersed work, relational complexity, and competing expectations while sustaining emotional availability (Poetz & Volmer, 2024). Examining leadership as it is experienced in practice is therefore essential to understanding whether, and under what conditions, it acts as a resource or a demand for leaders and team members.

We therefore argue that context-sensitive, qualitative research is needed to better understand the nuanced complexity of leadership in post-pandemic virtual and hybrid environments. Field-based studies, rather than experimental or lab-based designs, are especially vital for capturing how leadership is shaped by real-world constraints, relationships, and aspirations (Banker et al., 2023; Liao, 2017), and how it shapes well-being and effective team functioning over time. Given the role of leadership in shaping the well-being, functioning, and broader work outcomes of teams, and in contributing to a healthy organisational culture (Cortellazzo et al., 2019; Coulston et al., 2025a), this is a critical area for further enquiry.

### Research objectives

This exploratory study aims to enhance understanding of how leadership is experienced in virtual and hybrid team environments, and how these experiences relate to effective team functioning and well-being, from the perspectives of both leaders and team members. Specifically, it examines which leadership behaviours are most valued, how these are enacted or constrained in practice, and what tensions emerge when team and leader expectations diverge. To explore these questions, the study draws on a global sample spanning diverse working contexts, including healthcare, education, banking and professional services, across both public and private sector organisations.

The research adopts a relational lens to examine leadership as a socially embedded and dynamic process. Particular attention is given to how well-being emerges through leadership experiences, rather than being treated as a predefined outcome, particularly with regards to emotional, relational, and contextual demands. This framing is pertinent given that leadership can be experienced as a dual force: a source of meaning and motivation for teams, and simultaneously a site of emotional strain or depletion for leaders themselves (Poetz & Volmer, 2024).

This study contributes to the literature on leadership in three ways. First, it provides a qualitative account of leadership as lived and experienced by both leaders and team members in real-world virtual and hybrid contexts, highlighting the relational and everyday dimensions of leadership that are often less visible in survey-based or experimental research (Braun & Clarke, 2019; 2021; Braun & Clarke, 2006). Second, it examines leadership not only as a source of support, trust, and connection, but also as a site of emotional, relational, and contextual demand, thereby extending understanding of leadership in virtual and hybrid teams (Poetz & Volmer, 2024). Third, it highlights the tensions and misalignments that can arise between leadership aspirations, enacted leadership practices, and leader capacity in contemporary virtual and hybrid teams.

### Theoretical grounding

This study is grounded in a critical realist perspective, which conceptualises leadership as a socially embedded and relational phenomenon shaped through interaction between individuals, their context, and the structural conditions of work. Rather than viewing leadership as a fixed set of traits or behaviours, this perspective foregrounds leadership as a dynamic process that unfolds through everyday practices and relationships. This framing is particularly relevant in virtual and hybrid team contexts, where leadership must be enacted with reduced physical proximity and limited informal cues (Badrinarayanan, 2024).

To interpret how leadership is experienced and enacted in these settings, the study draws on the Job Demands-Resources (JD-R) model (Bakker & Demerouti, 2007​​​​,​​​ 2017). The JD-R model distinguishes between job demands, which require sustained effort and are associated with physiological and psychological costs, and job resources, which support people in managing those demands and achieving valued goals. In this study, the JD-R model is used as an interpretive framework rather than a predictive model, supporting examination of how leadership is experienced as supportive, demanding, or at times both, within virtual and hybrid team contexts.

Within the JD-R literature, leadership has been conceptualised in multiple ways, including as a job resource, a job demand, and a configuring influence on job demands, resources, and well-being (Bakker & Demerouti, 2017; Bakker et al., 2023; Tummers & Bakker, 2021). From this perspective, leadership may be understood not only as a foundation of resources for employees and teams; such as enabling autonomy, fostering meaningful connections, and supporting learning, but also as a role that can expose leaders themselves to substantial job demands, including sustained emotional, cognitive, and relational effort (Mazzetti & Schaufeli, 2022; Poetz & Volmer, 2024). These dynamics may be particularly salient in virtual and hybrid contexts, where reduced physical visibility, reliance on technology-mediated communication, and different expectations reshape how demands and resources are experienced by both leaders and team members (Efimov et al., 2022; Hincapie & Costa, 2024; Rohwer et al., 2020).

To extend later interpretation of the findings, this study also draws on Conservation of Resources (COR) theory (Hobfoll et al., 2018). COR complements the JD-R model by helping to explain how strain may accumulate over time when key resources, such as emotional energy, time, or social support, are lost, threatened, or not replenished, and how access to resources may help sustain resilience and leadership capacity in virtual and hybrid contexts.

## Method

This study employed a qualitative research design, selected for its capacity to uncover insights into subjective experiences that are difficult to capture through quantitative methods (Hefferon et al., 2017). While the dataset was originally generated as part of a broader investigation into well-being in virtual and hybrid teams (Coulston et al., 2025a), this analysis focuses specifically on leadership as a socially embedded and dynamic process.

The study is grounded in a critical realist ontology, recognising that social phenomena such as leadership are both real and shaped through interaction, meaning making and context. From this perspective, leadership is not a fixed behaviour but is co-constructed through relational and structural dynamics. Reflexive thematic analysis (Braun & Clarke, 2021, 2006) was used to support the study’s exploratory aims by enabling an interpretive and iterative examination of patterned meaning across the dataset. This analytic approach aligns with the critical realist epistemological positioning by attending to participants’ lived experiences while recognising the role of social, organisational, and contextual influences in shaping those experiences. The JD-R model was used as an interpretive framework for understanding the developed themes, rather than as a fixed analytic structure.

To generate rich and multi-layered insights, we conducted semi-structured interviews and focus groups. These methods elicited detailed accounts of participants’ lived experiences and explored how leadership was enacted and experienced across different virtual and hybrid team settings, highlighting points of both convergence and divergence.

### Ethics

Ethical approval was granted by the Psychiatry and Psychology PNM Research Ethics Panel, King’s College London, (Ref: LRS/DP-21/22–26093). Data was collected between the 11th November 2021 - 16 March 2022. Participants provided written informed consent after receiving information about the study purpose, voluntary participation, and confidentiality. All transcripts were anonymised and securely stored with access restricted only to the research team.

### Participants

Data for this study were drawn from a broader qualitative dataset generated to explore experiences of well-being and performance in virtual and hybrid teams across multiple organisational contexts. The dataset included rich discussion of communication, leadership, team dynamics, and adaptation in dispersed work. One broader paper from this dataset has already been published, focusing on the emergence of team well-being through a JD-R lens (Coulston et al., 2025a). The present paper reports a distinct focused secondary analysis of the dataset, with a specific analytic focus on how leadership was experienced and enacted in virtual and hybrid teams from the perspectives of both leaders and team members. The current analysis examined leadership behaviours, expectations, communication and support from both leader and team member perspectives.

The inclusion criteria, recruitment procedures, and data collection methods are reported in detail in Coulston et al. (2025a). This included:

- Up to four teams from any organisation utilising virtual and hybrid working, to ensure we captured a diversity of teams.
- Participants aged 18+  years, working at least two days a week remotely from the office as part of a defined team.
- Within each team, a minimum of three team members were required for each 60-minute semi-structured focus group, to ensure we were interviewing a group from a team. This excluded the team leader.
- Separate 60-minute semi-structured interviews were conducted with the team leader of each team interviewed.
- All participants to have fluency in English and be able to participate via the social networking site of Zoom or MS Teams.

Recruitment was facilitated through an industry partner and the research team’s professional networks, enabling access to a wide range of organisations across industries and geographies. Invitations were distributed via email, accompanied by an information sheet and consent form. Participation was voluntary, with the option to withdraw up to three months post-interview. All participants provided written informed consent.

The final dataset comprised 140 participants globally. This included thirty team leaders (interviewed individually in semi-structured interviews), and twenty-nine corresponding team focus groups (*n*  =  110 team members), interviewed in semi-structured team focus groups. These teams operated in virtual and hybrid working models and were drawn from both public and private sector organisations, including healthcare, academia, human resources, professional services, and banking.

Tables I and II provide a detailed overview of participant demographics. Participants were drawn from nine different private sector industries, including Banking (23%), Professional services (17%), Financial services (13%), Consultancy (10%), as well as public sector Education (10%), Healthcare (7%) and Not for Profit (7%). Roles spanned multiple levels, encompassing team members in operational, administrative, managerial and director roles, as well as team leaders from Manager to Managing Director, enabling a comprehensive exploration of leadership across hierarchical levels and job functions.

**Table I.** Demographics of team leaders.

Team leader	Gender	Age	Ethnicity	Role within Organisation	Location of Leader	Industry	Number of days/week usually working virtually at time of interview
1	Female	35−44	White	Senior Manager	United Kingdom	Education	3 to 4
2	Female	35−44	White	Director	United Kingdom	Banking	3
3	Female	35−44	White	Director	Ireland	Professional services	3 to 4
4	Female	25−34	Arabic	Senior Manager	United Kingdom	Healthcare	4 to 5
5	Male	35−44	Asian	Senior Manager	India	Professional services	5
6	Male	45−54	White	Executive	Netherlands	Professional services	2 to 2 1/2
7	Male	45−54	White	Director	South Africa	Consultancy	2 to 3
8	Male	45−54	White	Senior Manager	United Kingdom	Education	3
9	Female	35−44	White	Director	United Kingdom	Professional services	4 to 5
10	Female	45−54	White	Senior Manager	Italy	Human Resources	3
11	Female	35−44	White	Senior Manager	Netherlands	Consultancy	5
12	Female	45−54	White	Executive	United Kingdom	Not for Profit	1
13	Female	45−54	White	Director	United Kingdom	Healthcare	4 to 5
14	Female	45−54	White	Director	United Kingdom	Technology	4 to 5
15	Female	25−34	White	Executive	Europe/Caribbean	Not for Profit	4 to 5
16	Female	35−44	White	Manager	United Kingdom	Education	4
17	Female	45−54	White	Senior Manager	Sweden	Human Resources	5
18	Male	45−54	White	Managing Director	United Kingdom	Banking	2 to 3
19	Female	25−34	White	Manager	United States	Consultancy	4
20	Female	35−44	White	Director	United States	Consultancy	4 to 5
21	Male	45−54	White	Executive	France	Professional services	2 to 3
22	Male	45−54	White	Manager	United Kingdom	Banking	4 to 5
23	Female	35−44	Asian	Senior Manager	China	Banking	2 to 3
24	Male	45−54	White	Managing Director	United Kingdom	Banking	5
25	Male	45−54	Asian	Managing Director	India	Banking	5
26	Male	35−44	White	Managing Director	Hong Kong	Banking	mixed
27	Female	35−44	White	Senior Manager	Spain	Financial services	2 to 3
28	Female	25−34	White	Manager	United Kingdom	Financial services	5
29	Male	55−64	White	Executive	Australia	Financial services	2 to 3
30	Female	45−54	White	Director	United States	Financial services	mixed

**Table II.** Demographics of teams.

Team	Number of Team members in focus group	of which male	of which female	Location of Team	Industry	Number of days/week usually working virtually across team at time of inteview
1	3	0	3	United Kingdom	Education	3 to 4
2	3	1	2	Global	Banking	5
3	3	1	2	Ireland	Professional services	4 to 5
4	3	1	2	United Kingdom	Healthcare	4 to 5
5	4	3	1	India	Professional services	4 to 5
6	4	3	1	Netherlands	Professional services	3 to 5
7	3	0	3	South Africa	Consultancy	4 to 5
8	3	1	2	United Kingdom	Education	3 to 5
9	4	2	2	United Kingdom	Professional services	3 to 5
10	4	3	1	Europe	Human Resources	2 to 5
11	4	2	2	Netherlands	Consultancy	3 to 4
12	4	0	4	United Kingdom	Not for Profit	2 to 3
13	3	0	3	United Kingdom	Healthcare	4
14	3	1	2	United Kingdom	Technology	4 to 5
15	4	1	3	Global	Not for Profit	4 to 5
16	4	1	3	United Kingdom	Education	0 to 4
17				no data		
18	5	4	1	United Kingdom	Banking	3
19	4	2	2	United States	Consultancy	4 to 5
20	5	2	3	United States	Consultancy	4 to 5
21	3	3	0	France	Professional services	3 to 4
22	6	3	3	United Kingdom	Banking	3 to 4
23	4	0	4	China	Banking	2
24	4	2	2	Global	Banking	2 to 4
25	3	1	2	India	Banking	5
26	4	3	1	Hong Kong	Banking	4 to 5
27	5	4	1	Spain	Financial services	4 to 5
28	3	1	2	United Kingdom	Financial services	5
29	4	3	1	Global	Financial services	1 to 5
30	4	2	2	United States	Financial services	5
**Total**	**110**	**50**	**60**			

Geographically, the study included participants from over thirteen countries, with 38% of teams from the United Kingdom, 21% from across Europe, 14% from Asia, 14% from other global locations, as well as 10% from the United States and 3% from South Africa.

### Procedure

The semi-structured interviews and focus groups were conducted virtually via MS Teams or Zoom, each lasting approximately 60 minutes. Interview and discussion guides were tailored separately for team leaders and team members, enabling exploration of distinct perspectives while maintaining alignment through a shared thematic structure.

For both groups, topic guides focused on connection, communication, leadership behaviours, well-being, performance and adaptation within virtual and hybrid work environments. Leadership consistently emerged as an important theme across the interviews and focus groups. Participants were invited to reflect on their own experiences and expectations of leadership in relation to virtual and hybrid work.

Team leaders were asked questions such as:

*“In what ways do you communicate as a leader with your team?”*

*“In a virtual or hybrid team environment, how do you lead? Are there differences to how you would lead in-person teams? If so what?”*

Team members were asked questions such as:

*“How would you describe how you are led as a team, by your team leader?”*

*“In a virtual or hybrid team environment, how would you like to be led?”*

### Analysis

All interviews and focus groups were transcribed verbatim, either by a professional transcription service (*n* = 25) or a member of the research team (*n* = 34). Identifiable details, including names and organisational affiliations, were removed to ensure confidentiality. NVivo 15 software (QSR, 2018) supported data organisation, coding, and analysis.

Reflexivity was treated as integral to the analytic process. The analysis was led by the first author (CC), who engaged in ongoing reflexive practice throughout familiarisation, coding, and theme development, including memo-writing and iterative reflection on how prior professional experience, research positioning, and assumptions about leadership may have shaped interpretive decisions. Themes were generated inductively through close engagement with the data, with attention to patterned meaning across participants’ accounts.

**Figure 1.**
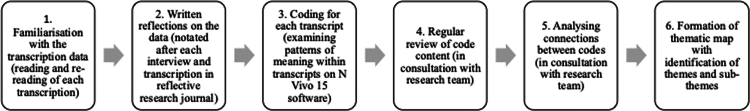
Thematic analysis six-stage process in accordance with Braun and Clarke (2006).

The analysis followed the six phases of Braun and Clarke’s reflexive approach to thematic analysis (Braun & Clarke, 2006; Clarke & Braun, 2017), and is visualised in Figure 1. This approach supported the development of themes grounded in participants’ lived experiences. Initial coding, led by the first author (CC), focused on how leadership was described, experienced, and valued by both leaders and team members. Codes were iteratively developed and organised into broader categories reflecting team aspirations, enacted leadership behaviours, and leadership-related tensions, in alignment with the study’s aims.

The JD-R model was used as an interpretive framework for understanding the developed themes, rather than as a fixed analytic structure or a pre-defined coding frame. Collaborative discussions within the research team were used to support reflexive sense-making, challenge interpretations, consider alternative readings of the data, and refine theme boundaries.

### Reflexivity statement

This study was developed by the first author (Cass Coulston), as part of a postgraduate research programme examining factors influencing leadership, well-being, and performance in virtual and hybrid teams. The research was designed to examine the perspectives of both team members and leaders, with the intention of informing subsequent quantitative research phases.

The first author brings prior professional experience as both a team leader and a senior practitioner working with teams across organisational contexts. This background provided familiarity with the challenges of leadership and collaboration in complex work environments, while also necessitating careful reflexive attention to how prior assumptions, professional identity, and experiential knowledge might shape interpretation of what participants described as effective, supportive or demanding leadership.

CC has had direct supervision from MD and RT through the process, who also have an interest in well-being and have previously published research on workplace stress and interventions to improve well-being at work. SS has published extensive research on social cognition, understanding the factors influencing social interaction, and impacts on mental health. Ongoing dialogue with the supervisory team supported reflexive sense-making, challenged interpretations, considering alternative explanations, and refining the meaning and boundaries of the themes.

## Results

**Figure 2.**
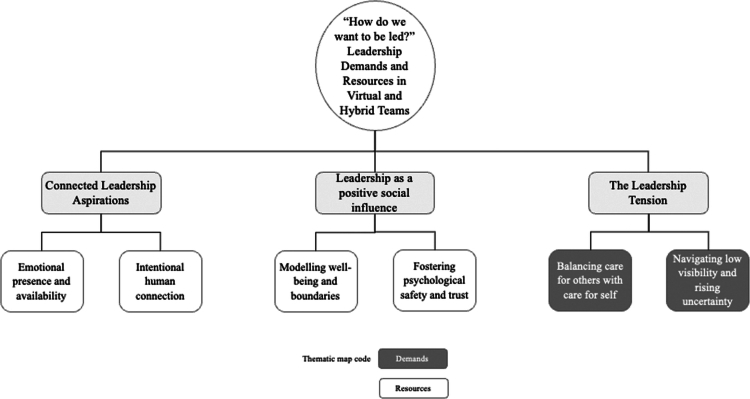
Thematic map of overarching themes and sub-themes.

Thematic analysis of the interviews and focus groups generated three overarching themes that captured how leadership was described, experienced, and valued in virtual and hybrid teams. These themes: “Connected Leadership Aspirations,” “Leadership as a Positive Social Influence” and “The Leadership Tension,” illuminated the multi-faceted nature of leadership, highlighting both the expectations placed on leaders and the relational value leaders bring. Each theme comprised two sub-themes that captured nuanced patterns across leader and team member perspectives. Figure 2 presents a thematic map, while Table III provides a further overview of the themes and sub-themes with illustrative quotes. The following sections explore each theme in turn, drawing on participants’ voices to illustrate convergences and tensions in how leadership was enacted, experienced, and sustained.

**Table III.** Summary table of results.

Overarching Themes	Sub-Themes	Illustrative Quotes
**Connected Leadership Aspirations**	Emotional presence and availability	*You know it's recognising that things are slightly different so you may have to put a bit more effort in and reassert and reaffirm that “I'm available”. You know, the thing about my door is always open, that's a physical thing…but what do you do to create that sense when you're working virtually. And trying to do that, is what makes a difference. When you know that your leader cares and is trying to do that, that makes a difference.* Team member 3, Team 13, Healthcare, UK. *I guess we just want… and they continue to show that confidence in us. And, we basically continue to feel that, Okay, if you need any help, they are there, available through multiple channels to multiple modes. And we can easily reach out to them, so that we don't feel left alone at any point of time.* Team member 1, Team 5, Professional Services, India. *I think it is about being as honest as you can be and as transparent as you can be around how you are feeling, where you're at, what you're thinking about, and I think that's important as a leader. And you know, I will turn up sometimes to a meeting in the morning, and I will be exhausted, because kids have been up during the night or something. And I try to be honest about that, because I think that sharing that vulnerability, sharing that not everything is always rosy and happy, is important for everybody to understand and other people will share those things too.* Team leader, Team 14, Technology, UK.
	Intentional human connection	*I think from a leadership point of view, it’s very isolating if you don’t have connection, if you don’t make your team feel right, respecting their life, or show that you value their time. I think that if you’re not intentional about that, you risk discouraging people. In an office, you might say hi or grab coffee. But now you have to create that space intentionally - private time to check in and show people they matter.* Team member 2, Team 9, Professional Services, UK. *For me, connect means making people feel that we have a strategy, we have a strategy, not me. We have a strategy. We are here in this constant change, and we are okay. We can change. We can do things, we can experiment, we can connect just to have a cup of tea, and we can connect to design the strategy.* Leader, Team 10, Human Resources, Europe. *Also turning the mirror on myself, being so busy, and, you know, I like to think I'm available, but my agenda is not really that available if I look at it, right. So, you know, the availability of each other and the leaders is another…. So I think what hinders that sense of belonging and that connectiveness is you know, the other work.* Team leader, Team 11, Consultancy, Netherlands.
Overarching Themes	Sub-Themes	Illustrative Quotes
**Leadership as a positive social influence**	Modelling well-being and boundaries	*I think if I don't see the leaders on my project, taking breaks to go and walk during the day, or do things like that, then I'm less inclined to do that, because I think I have to be on. So you know, by seeing them set examples like that and really follow through, I feel more inclined and empowered to do those things. So I think it helps when it comes from the top and you see your leaders - they don't just say, oh you have to take care of your wellbeing, they're actually doing things for their own wellbeing. And so that hits home for me and makes me realise, Okay I need to do that as well. And I feel comfortable to do that because this truly is a team and a leadership team that values that.* Team member 1, Team 19, Consultancy, US *I think there is an element of leadership by example as well…I know that the leadership team were all told, you need to start taking a bit of holiday, show the team that you need to look after your well-being, you need to look after yourself and get rest. So those sorts of ways, that it's not just saying - this is the right thing to do - but showing that it is as well are important.* Team member 3, Team 24, Banking, China *I think it's a completely different skill set…. So this is kind of challenging leaders to rethink the way that they have been leading. What works? What doesn't work?.... if you're holding on trying to do what you used to do and still keep doing it that way, it's going to be even harder. I would say it probably is challenging, and that leadership in this hybrid world is more important than it has ever been.* Leader, Team 3, Professional Services, Ireland
	Fostering psychological safety and trust	*So the thing that I need to let go of is a very old fashioned arbitrary, socially constructed, what the hour, what the day needs to look like. What I need to hold onto more, so that I can be more effective in my role, is that I trust my colleagues to get on with the work, to deliver it at a high standard, to deliver it on time and everything else doesn't matter.* Leader, Team 4, Healthcare, UK *There are some managers who probably want to have the face time. The fact that they need to see you sitting at your desk no matter what you're doing…but they just want to see you. It's that element of trust and if they've given you a task, they know that you're gonna follow through, finish it, get on with it. So that in itself is extremely powerful. And I definitely don't take it for granted. Because I've met many teams that are not like that. And the micromanaging actually is detrimental for the team.* Team member 3, Team, 24, Banking, China *It really depends on the individual, but I'd say for me, I don't need to be micromanaged…if I was to have people constantly checking on me to make sure I'm working. I mean, I don't work well under those circumstances. It would lead me to change my job. But thankfully and luckily I haven't actually encountered that at (name of company). I’ve been quite happy with the line managers that I've had.* Team member 2, Banking, Hong Kong
Overarching Themes	Sub-Themes	Illustrative Quotes
**The Leadership Tension**	Balancing care for others with care for self	*Sometimes I think maybe I should be checking more. But then other times, on the other hand, I think, I can't be checking everything, because it's not healthy for me and not even for the person. We're not micromanaging people here. I need to trust them. But then the backlash comes to me. And then at this point, I think I should have checked more. So it's always a bit of this tension.* Leader, Team 1, Education, UK. *But as a leader, a lot of times I am so inundated with people coming to me with requests to get to know me, requests for my time, especially with so many people being new, that I sometimes forget that I could make that same level of effort, because in my mind, there's so much else going on, and there's so many people that are seeking me out.* Team leader, Team 20, Consultancy, US *So I get worried, is it too much work they have? Is it that they just can't turn off and there is a bigger problem emotionally or in in their head that needs to be addressed to help them switch off? Or am I not giving them enough support, so they have to work in the evenings?...It puts me in the anxiety mode of is everything actually OK? Or are they just saying it’s OK and don't want to talk to me?…then I just seem to be working and sitting here all day long and all night long trying to make sure everything is under control…it puts me into this overload and overwork.* Team leader, Team 16, Education, UK.
	Navigating low visibility with rising uncertainty	*Well, I think it's important to be able to read them and lead them properly and be able to see if something is not working. Also when you're sitting in the office, you observe more….and at home for example, I’m not hearing it, but when I'm in the office and they're in their office, I do pick up stuff. So whereas when you're working remotely, you can only see what they actually let you see and what they tell you. Not that they're trying to hide anything, but it's natural, right? Whereas when they're in the office, you get to know the staff more. And you see how they behave, and you can coach them a bit more.* Leader, Tam 27, Financial services, Spain. *I like bouncing ideas off people. I thrive on being in a room. I think I'm quite good at picking up on cues and picking up on body language and reading a room. And I found that quite hard to start with, particularly working with a new client, working with a new team. So I think it's about learning people's behaviours online and just learning how to pick up on the cues.* Leader, Team 9, Professional Services, UK. *They need to learn to understand a bit more, right, what to try. If they can step in the shoes of their employees…. So I think that already for performance management, it's difficult to work in a virtual setting because your manager would not be around sitting next to you, seeing how you talk to different people. I mean some of them are different or just old school, right. So they still look at how long have you worked in a day. Obviously that won’t work in this…so they need to think more holistically.* Team member 3, Banking, Hong Kong

### Theme one: connected leadership aspirations

This overarching theme, “Connected Leadership Aspirations,” reflected a consistent and salient desire, expressed by both team members and leaders, for leadership grounded in meaningful interpersonal connection. Across the dataset, participants described effective leadership in virtual and hybrid contexts as extending beyond task coordination to encompass the relational and emotional needs of individuals.

This conception of leadership contrasted more transactional models, with participants describing connection as fundamental to fostering well-being, cohesion, and a sense of psychological safety. The aspiration for such relational leadership was shared across roles, surfacing most strongly in contexts of reduced visibility and high levels of complexity or demand. Two sub-themes, “Emotional Presence and Availability” and “Intentional Human Connection,” illustrated how this aspiration was experienced and expressed, showing connection as both an emotional state and a relational practice.

#### Sub-theme one: emotional presence and availability

This sub-theme captured the relational nature of leadership that both team members and leaders valued. Availability was not simply logistical but experienced through empathy, emotional presence, and human care. Rather, team members expressed a desire for leaders who were emotionally available, attentive, and responsive, not only in coordinating work, but in acknowledging the human experience behind the tasks. Emotional presence was viewed as a source of trust and reassurance, particularly when visibility was low and isolation risked creeping in.

*You know it's recognising that things are slightly different so you may have to put a bit more effort in and reassert and reaffirm that “I'm available”. You know, the thing about my door is always open, that's a physical thing…but what do you do to create that sense when you're working virtually. And trying to do that, is what makes a difference. When you know that your leader cares and is trying to do that, that makes a difference.* Team member 3, Team 13, Healthcare, UK.

Connection was understood as something leaders actively constructed; not a passive outcome of proximity, but a relational practice requiring intention. Leaders who showed up in small, human ways, by checking in, being honest about their own challenges, and being reachable, were remembered and valued.

*I guess we just want… and they continue to show that confidence in us. And, we basically continue to feel that, Okay, if you need any help, they are there, available through multiple channels to multiple modes. And we can easily reach out to them, so that we don't feel left alone at any point of time.* Team member 1, Team 5, Professional Services, India.

Leaders acknowledged that expectations had shifted since the widespread transition to virtual and hybrid work. Availability was no longer about open-door policies or corridor conversations; it demanded a relational presence that blurred personal and professional boundaries. Many described creating space for informal dialogue, showing emotional openness, and modelling authenticity to foster trust and psychological safety.

*I think it is about being as honest as you can be and as transparent as you can be around how you are feeling, where you're at, what you're thinking about, and I think that's important as a leader. And you know, I will turn up sometimes to a meeting in the morning, and I will be exhausted, because kids have been up during the night or something. And I try to be honest about that, because I think that sharing that vulnerability, sharing that not everything is always rosy and happy, is important for everybody to understand and other people will share those things too.* Team leader, Team 14, Technology, UK.

Yet, this availability carried a cost. Several leaders reflected on the emotional labour involved and the tension between wanting to be available and the realities of workload and time pressure. Emotional presence and availability emerged as central to connected leadership; an important relational resource, but also a fragile and effortful practice.

#### Sub-theme two: intentional human connection

While the first sub-theme focused on emotional presence and relational availability, this second sub-theme captured the proactive and deliberate efforts leaders made to create human connection in dispersed work settings. In hybrid and virtual environments, where informal encounters were often absent, fostering team togetherness and belonging required conscious design and adaptation.

*I think from a leadership point of view, it’s very isolating if you don’t have connection, if you don’t make your team feel right, respecting their life, or show that you value their time. I think that if you’re not intentional about that, you risk discouraging people. In an office, you might say hi or grab coffee. But now you have to create that space intentionally - private time to check in and show people they matter.* Team member 2, Team 9, Professional Services, UK.

Leaders described becoming increasingly intentional in shaping opportunities for connection through shared rituals, informal check-ins, and inclusive communication practices. These were not perceived as optional extras, but as essential mechanisms for sustaining connection, trust, cohesion and belonging.

*For me, connection means making people feel that we have a strategy, we have a strategy, not me. We have a strategy. We are here in this constant change, and we are okay. We can change. We can do things, we can experiment, we can connect just to have a cup of tea, and we can connect to design the strategy.* Leader, Team 10, Human Resources, Europe.

Team members echoed the value of deliberate relational time, noting that even brief one-on-one conversations signalled care and commitment. Both leaders and team members emphasised the need to diversify communication methods, adapting language, format, and channels, to increase accessibility and emotional resonance across locations and time zones. However, these efforts were not without cost. Many leaders described rethinking team rhythms to create space for connection while avoiding meeting fatigue. Others acknowledged that when pressures mounted, relational leadership was often the first to be compromised.

*Also turning the mirror on myself, being so busy, and, you know, I like to think I'm available, but my agenda is not really that available if I look at it, right. So, you know, the availability of each other and the leaders is another…. So I think what hinders that sense of belonging and that connectiveness is you know, the other work.* Team leader, Team 11, Consultancy, Netherlands.

Taken together, this sub-theme highlighted how deliberate relational practices, such as inclusive dialogue, shared rituals, and regular check-ins served as motivational resources in virtual and hybrid work. When neglected or difficult to sustain, however, they risked becoming additional demands, contributing to leader strain and reduced team cohesion.

### Theme two: leadership as a positive social influence

This second overarching theme captured how leaders shaped the emotional climate and team culture through their everyday behaviours. Participants described how leaders who modelled healthy boundaries, prioritised well-being, and fostered psychologically safe spaces positively influenced how others felt and worked. Leadership was not only about direction or performance; it was about shaping the tone of interaction, how people related to one another and engaged with their work.

At the same time, many leaders reflected on the difficulty of consistently enacting these behaviours under pressure, even when they recognised their importance. This theme was developed through two interrelated yet distinctive sub-themes: “Modelling Well-being and Boundaries” and “Fostering Psychological Safety and Trust.”

#### Sub-theme one: modelling well-being and boundaries

This sub-theme explored how leaders influenced team culture by modelling healthy boundaries and well-being practices, both implicitly and explicitly. In virtual and hybrid contexts where work-life boundaries often blurred, leaders who visibly prioritised their own well-being sent an empowering message to their teams. Some team members described a shift from performative encouragements toward more authentic, observable actions. Leaders who took regular breaks, used their full leave allowance, or shared personal well-being routines were seen as enabling and inspiring. Such examples helped normalise behaviours such as pausing during the workday, working flexibly, and prioritising family or physical health.

*I think if I don't see the leaders on my project, taking breaks to go and walk during the day, or do things like that, then I'm less inclined to do that, because I think I have to be on. So you know, by seeing them set examples like that and really follow through, I feel more inclined and empowered to do those things. So I think it helps when it comes from the top and you see your leaders - they don't just say, oh you have to take care of your wellbeing, they're actually doing things for their own wellbeing. And so that hits home for me and makes me realise, Okay I need to do that as well. And I feel comfortable to do that because this truly is a team and a leadership team that values that.* Team member 1, Team 19, Consultancy, US.

This influence extended to respecting working hours, modelling digital boundaries, and encouraging rest or the use of support resources. In some teams, coaching had been used to help individuals identify what energised or depleted them, with leaders taking a facilitative role in helping people set sustainable boundaries.

*I think there is an element of leadership by example as well…I know that the leadership team were all told, you need to start taking a bit of holiday, show the team that you need to look after your well-being, you need to look after yourself and get rest. So those sorts of ways, that it's not just saying - this is the right thing to do - but showing that it is as well are important.* Team member 3, Team 24, Banking, China.

Nonetheless, participants also highlighted the pressures leaders faced in maintaining these behaviours, particularly under mounting workloads. Some described the challenge of ‘living at work,’ needing to self-manage digital boundaries in the absence of physical cues. Others spoke of the internal pressure to remain visible and constantly available, even when recognising this as unsustainable. Across interviews, leaders reflected on the need to rethink familiar ways of leading, acknowledging how virtual and hybrid contexts demanded new skills and mindsets:

*I think it's a completely different skill set…. So this is kind of challenging leaders to rethink the way that they have been leading. What works? What doesn't work?.... if you're holding on trying to do what you used to do and still keep doing it that way, it's going to be even harder. I would say it probably is challenging, and that leadership in this hybrid world is more important than it has ever been.* Leader, Team 3, Professional Services, Ireland.

Taken together, this sub-theme illustrated how leaders served as both role models and boundary setters, influencing their wider climate of well-being and engagement within teams. When enacted deliberately, such behaviours operated as a form of positive social influence; a motivational and protective resource. When constrained or absent, they revealed the tensions leaders faced in sustaining well-being practices amid the complexities of virtual and hybrid work.

#### Sub-theme two: fostering psychological safety and trust

This sub-theme highlighted the importance of trust and psychological safety as core elements of team effectiveness and well-being in virtual and hybrid contexts. Participants described how leaders fostered a sense of safety not through oversight or visibility, but by demonstrating trust in their teams’ professionalism and enabling people to work in ways that suited them best.

A consistent message across teams, recognised by both leaders and team members, was that micromanagement, often viewed as a legacy of traditional leadership norms, undermined motivation and performance. In contrast, leaders who role-modelled trust, focusing on outputs rather than monitoring activity, were perceived as empowering and progressive. Some leaders reflected on the need to consciously release outdated notions of the ‘working day,’ shifting instead toward a values-based, outcome-focused approach.

*So the thing that I need to let go of is a very old fashioned arbitrary, socially constructed, what the hour, what the day needs to look like. What I need to hold onto more, so that I can be more effective in my role, is that I trust my colleagues to get on with the work, to deliver it at a high standard, to deliver it on time and everything else doesn't matter.* Leader, Team 4, Healthcare, UK.

Psychological safety was reinforced when leaders gave their teams autonomy and respected different working styles. The presence of trust was widely described as energising by team members, while its absence, particularly in the form of unnecessary supervision, was viewed as detrimental.

*There are some managers who probably want to have the face time. The fact that they need to see you sitting at your desk no matter what you're doing…but they just want to see you. It's that element of trust and if they've given you a task, they know that you're gonna follow through, finish it, get on with it. So that in itself is extremely powerful. And I definitely don't take it for granted. Because I've met many teams that are not like that. And the micromanaging actually is detrimental for the team.* Team member 3, Team, 24, Banking, China.

For many, trust-based leadership was a facilitator of performance and a factor in retention. Several participants noted that leaders who avoided excessive oversight enabled them to thrive. Across teams, these accounts revealed that psychological safety was more than a cultural aspiration; it was enacted through specific leader behaviours. Leaders who trusted their teams to deliver without constant visibility, and who encouraged open dialogue about effective ways of working and aligned accountability were perceived as enabling a more human, effective, and resilient way of working.

*It really depends on the individual, but I'd say for me, I don't need to be micromanaged…if I was to have people constantly checking on me to make sure I'm working. I mean, I don't work well under those circumstances. It would lead me to change my job. But thankfully and luckily I haven't actually encountered that at (name of company). I’ve been quite happy with the line managers that I've had.* Team member 2, Banking, Hong Kong.

### Theme three: the leadership tension

This final overarching theme centred on the leader’s evolving experience of virtual and hybrid work, which often involved holding space for others, while navigating new dynamics and absorbing invisible demands. Many leaders described a strong desire to support their teams amidst rising responsibilities and fewer informal opportunities to recharge.

Two sub-themes emerged. The first, “Balancing care for others with care for self,” reflected the inner tension leaders experienced in striving to be available, empathetic, and responsive, even when their own capacity was stretched. The second, “Navigating low visibility and rising uncertainty,” captured the disruption to familiar feedback loops and the challenge of maintaining connection and oversight in more distributed settings. While these shifts created moments of disconnection, they also prompted reflection and a growing commitment among leaders to adapt, stay present, and lead with greater intentionality.

#### Sub-theme one: balancing care for others with care for self

This sub-theme captured the internal tension leaders experienced as they sought to care for and remain connected to their teams while also managing their own workload, energy, and well-being. Leaders described the emotional labour involved in maintaining team morale and psychological safety in virtual and hybrid settings, often without the informal cues and spontaneous interactions that once helped them gauge how people were coping. Many felt the burden of being constantly available, absorbing invisible demands, and carrying responsibility for others’ well-being, sometimes at a cost to their own.

*Sometimes I think maybe I should be checking more. But then other times, on the other hand, I think, I can't be checking everything, because it's not healthy for me and not even for the person. We're not micromanaging people here. I need to trust them. But then the backlash comes to me. And then at this point, I think I should have checked more. So it's always a bit of this tension.* Leader, Team 1, Education, UK.

Several leaders reflected on how easy it was to overlook their own needs amidst ongoing requests for support, especially when striving to lead with compassion and responsiveness. Others described the cumulative pressure of trying to stay visible and approachable, while managing feelings of disconnection and concern for team members' well-being.

*But as a leader, a lot of times I am so inundated with people coming to me with requests to get to know me, requests for my time, especially with so many people being new, that I sometimes forget that I could make that same level of effort, because in my mind, there's so much else going on, and there's so many people that are seeking me out.* Team leader, Team 20, Consultancy, US.

This sense of imbalance was further compounded by the difficulty of interpreting team dynamics remotely. Without hallway chats, informal cues or non-verbal signals, leaders worried about how individuals were *really* doing and whether they were silently struggling. The resulting ambiguity created emotional strain and, at times, self-doubt.

*So I get worried, is it too much work they have? Is it that they just can't turn off and there is a bigger problem emotionally or in in their head that needs to be addressed to help them switch off? Or am I not giving them enough support, so they have to work in the evenings?...It puts me in the anxiety mode of is everything actually OK? Or are they just saying it’s OK and don't want to talk to me?…then I just seem to be working and sitting here all day long and all night long trying to make sure everything is under control…it puts me into this overload and overwork.* Team leader, Team 16, Education, UK.

Taken together, these accounts revealed a complex picture of virtual and hybrid leadership in which emotional care, presence, and self-boundary were often in tension. Leaders navigated new relational dynamics without the same feedback loops, often at a personal cost. The findings illuminated the *unseen labour* of leadership in virtual and hybrid environments, where holding space for others frequently meant stretching one’s own emotional and cognitive capacity.

#### Sub-theme two: navigating low visibility and rising uncertainty

Both leaders and team members wrestled with the challenge of collaborating and leading under conditions of reduced visibility. A clear tension emerged: most leaders described missing the subtle cues and spontaneous opportunities to guide or support their people, while some team members voiced concern about outdated assumptions, lack of trust, and unfair judgments when working remotely.

*Well, I think it's important to be able to read them and lead them properly and be able to see if something is not working. Also when you're sitting in the office, you observe more….and at home for example, I’m not hearing it, but when I'm in the office and they're in their office, I do pick up stuff. So whereas when you're working remotely, you can only see what they actually let you see and what they tell you. Not that they're trying to hide anything, but it's natural, right? Whereas when they're in the office, you get to know the staff more. And you see how they behave, and you can coach them a bit more.* Leader, Team 27, Financial services, Spain.

Many leaders described a loss of situational awareness, missing the informal moments when they could read a room, notice subtle shifts in mood or energy, or offer support in real time. The digital filter removed many of the micro-cues that once helped them steer, coach, or intervene early.

*I like bouncing ideas off people. I thrive on being in a room. I think I'm quite good at picking up on cues and picking up on body language and reading a room. And I found that quite hard to start with, particularly working with a new client, working with a new team. So I think it's about learning people's behaviours online and just learning how to pick up on the cues.* Leader, Team 9, Professional Services, UK.

From the perspective of some team members, however, this erosion of visibility sometimes translated into a sense of mistrust. Participants expressed frustration with assumptions that equated visibility with productivity. There was a perception that some leaders had struggled to fully adapt their mindset, holding onto older models of presenteeism rather than shifting toward outcome-based trust.

*They need to learn to understand a bit more, right, what to try. If they can step in the shoes of their employees…. So I think that already for performance management, it's difficult to work in a virtual setting because your manager would not be around sitting next to you, seeing how you talk to different people. I mean some of them are different or just old school, right. So they still look at how long have you worked in a day. Obviously that won’t work in this…so they need to think more holistically.* Team member 3, Banking, Hong Kong.

This friction between leaders’ need for assurance and team members’ need for autonomy highlighted the challenge of trying to sustain trust, connection, and oversight under conditions of reduced visibility and ongoing ambiguity.

## Discussion

Across the data, there was strong alignment between team members and leaders in their shared aspirations for leadership. Both valued human connection, psychological safety, and a more emotionally intelligent, relational approach; one that was attuned, available, and supportive in a virtual and hybrid context. The first two overarching themes of “Connected leadership aspirations” and “Leadership as a positive social influence” reflected this joint vision, but from different vantage points. The former captured the felt experience of connection and care, while the latter revealed how such values were enacted through behaviour. For instance, by modelling trust, setting boundaries, and fostering well-being. Together, they illustrated leadership as a relational resource that fostered belonging, mutual care, and cohesion across virtual and physical divides. These findings resonated with recent work showing that emotionally supportive leadership not only enabled psychological safety but also enhanced engagement and belonging in virtual and hybrid settings (Lechner & Tobias Mortlock, 2022; Pope & Miles, 2022).

However, while aspirations were often shared, the burden of enacting them appeared unequally carried. Leaders described a growing emotional load, feeling as though they held the team together, anticipated needs and role-modelled care. At the same time, leaders were navigating reduced visibility, rising ambiguity and feeling fewer opportunities for informal feedback or replenishment. The third overarching theme, “The Leadership Tension,” and its sub-themes of “Balancing care for others with care for self” and “Navigating low visibility and rising uncertainty,” captured this paradox. Leaders articulated a desire to show up with warmth and availability, yet simultaneously several leaders felt stretched and under-resourced. Leadership in virtual and hybrid contexts thus appeared as a double bind: the more one tried to be a relational resource for others, the more personally depleting the role could become, when organisational scaffolding was limited, emphasising its inherent complexity (Cortellazzo et al., 2019).

These findings extend recent scholarship that has questioned the assumption that leadership was purely a source of motivation or clarity for others (Poetz & Volmer, 2024). Instead, the findings suggest that leadership in virtual and hybrid teams may be experienced as both a resource and a demand, a dual dynamic that aligns with JD-R theory (Bakker & Demerouti, 2017, 2007). When leaders were supported organisationally, they appeared able to be energising and relationally supportive in dispersed work settings. When these conditions were absent, leadership itself could become a site of demand, amplifying the risk of emotional fatigue and strain. Recent research similarly suggests that leaders who engage in caring and relational behaviours may experience emotional exhaustion over time when such efforts are not adequately recognised, reciprocated, or supported (Handke & Wesche, 2024; Mazzetti & Schaufeli, 2022; Poetz & Volmer, 2024).

COR theory further deepened this interpretation by highlighting how sustained emotional demands could contribute to resource depletion over time (Hobfoll et al., 2018). Leaders in this study often continued to offer emotional support and care even while feeling depleted themselves. Over time, this may contribute to cumulative depletion, where behaviours experienced as leadership strengths for others begin to undermine leaders’ own well-being, as supported by the COR theoretical model. The expectation to ‘hold space’ for others, while having limited opportunities for their own recovery or processing, reflects what da Silva et al. (2022) described as the invisible weight of sensible leadership; a form of care that may be internalised, effortful, and insufficiently recognised.

To clarify the interconnections and distinctions among the overarching themes at an interpretive level, Table IV summarises how leadership was experienced across emotional, social and psychological dimensions and how these themes relate to the JD-R and COR framing.

**Table IV.** Interpretive summary of the interrelationship and distinction between the three overarching themes.

Theme	Core Focus	Primary dimension	Leadership Function	Interpretive relevance to JD-R/COR
Connected Leadership Aspirations	Desired qualities of connection and care	Emotional	Leadership as an emotional resource fostering trust, belonging, and safety	Emotional resource generation
Leadership as a Positive Social Influence	Enactment of care through daily behaviours	Social	Leadership as a social resource promoting cohesion and motivation	Motivational (resource-building) pathway
The Leadership Tension	Emotional and cognitive strain of sustaining care	Psychological	Leadership as a psychological demand under reduced visibility and support	Health-impairment (resource depleting) pathway

Importantly, these dynamics were not simply a matter of individual resilience or emotional intelligence. Rather, they pointed to leadership as a co-constructed process in virtual and hybrid teams. Team members shaped leadership not only through their needs and expectations, but also through their responsiveness, engagement, and feedback. Leaders, in turn, adjusted their behaviours in response to these relational cues, often increasing communication or support when they sensed uncertainty or low morale. This interactive loop suggests that leadership in dispersed work is best understood as a dynamic exchange of emotional, social, and structural resources rather than as a one-way set of leader behaviours.

Leadership behaviours therefore appeared to activate either supportive or depleting pathways depending on the alignment between team expectations, leader capacity, and contextual support. When aspirations were mutually recognised and supported, relational leadership appeared to foster trust, motivation, and cohesion, consistent with the motivational pathway of JD-R (Bakker & Demerouti, 2024). When these aspirations were unsupported or misaligned, the same behaviours could become emotionally straining, reflecting the health-impairment pathway of JD-R and the depletion dynamics described in COR theory (Bakker & Demerouti, 2024; Hobfoll et al., 2018). These patterns highlight the systemic and relational nature of leadership outcomes in virtual and hybrid contexts.

Additionally, findings nuanced the concept of availability in leadership. Emotional presence, being attuned, consistent, and open, emerged as both central and fragile. Leaders who communicated with calm and care fostered trust and cohesion (Hughes & Saunders, 2021; Lu, 2015), yet this form of presence was effortful, particularly without informal cues or feedback loops. Visibility, both in terms of being seen and being able to see, was experienced as a double-edged sword. Reduced visibility created uncertainty for leaders, while for team members, it shaped perceptions of fairness and trust. Some expressed concern that leaders were reverting to outdated models of presenteeism, judging contribution by online activity rather than outcomes. These tensions echoed earlier findings about the risks of misalignment and psychological distance in virtual settings (Coulston et al., 2025a).

Taken together, these findings extended JD-R by showing that leadership in virtual and hybrid teams was not a static input but a relationally fluctuating phenomenon that could operate as both support and strain depending on structural and emotional conditions. They also suggested that, from a COR perspective, shared aspirations for connected leadership could become difficult to sustain when they were not adequately supported or replenished, contributing to cycles of emotional depletion over time.

More broadly, the findings contribute to a growing literature on sustainable leadership by illuminating the often unseen cost of care and the need to design leadership environments that protect as well as enable (Lechner & Tobias Mortlock, 2022). Connected leadership remained a powerful aspiration in virtual and hybrid work, but these findings suggested that it must also be resourced structurally, relationally, and emotionally if it was to remain sustainable over time.

### Practical implications

The study findings highlight a need to reframe how organisations design, resource, and sustain leadership in virtual and hybrid environments. Leadership in these contexts is not only a performance function but also a relational and emotional practice that depends on systemic support (Costa et al., 2024; Zheng et al., 2024). To sustain leadership, organisations must move beyond expecting individual leaders to carry the full responsibility for connection and care, towards creating conditions in which connection is shared and co-produced across levels of the system (Pope & Miles, 2022).

At an organisational level, emotionally present and responsive leadership cannot be maintained through performance metrics or goodwill alone. Leadership capacity should be treated as a shared resource requiring active replenishment. This involves embedding regular opportunities for reflection, peer learning, and coaching, where leaders can share experiences and restore emotional energy. Aligning expectations with capacity is also essential. Workload, role design, and hybrid norms must support rather than contradict aspirations for relational and compassionate leadership. When organisations model well-being through their systems, by recognising the value of rest, boundaries, and psychological safety, they signal that care and sustainability are integral to effective leadership rather than signs of limitation.

At a leadership level, the findings underline the importance of balancing relational presence with psychological boundaries. The most effective virtual and hybrid leaders are those who lead with empathy and availability while also modelling sustainable practices, communicating limits clearly, prioritising recovery, and demonstrating authenticity in their approach to care (Hughes & Saunders, 2021; Mazzetti & Schaufeli, 2022). Leadership development programmes should therefore address not only technical competence but also the emotional labour of leadership, incorporating practices that strengthen emotional literacy, boundary-setting, and relational intelligence (Morrison-Smith & Ruiz, 2020). Regular feedback loops that help leaders understand how their presence and communication are experienced within teams can also reduce uncertainty and strengthen trust in conditions of low visibility (Coulston et al., 2025a).

At a team level, leadership should be seen as co-constructed rather than delivered. Teams that actively share the work of connection, expressing appreciation, engaging in open communication, and demonstrating mutual care, help reduce over-dependence on the leader as the sole source of motivation or support (Hill & Bartol, 2016; Wei et al., 2018). Creating shared norms for reflecting on what sustains or depletes collective energy, and encouraging upward care towards leaders, helps to distribute the emotional load and foster a more resilient, psychologically safe climate (Demerouti, 2023; Urien et al., 2021). When responsibility for well-being and connection is shared, care becomes mutual rather than hierarchical, making it more sustainable over time.

For Human Resources and leadership development functions, these findings also call for a shift. Programmes that have traditionally focused on individual competencies or outputs now need to address the emotional and systemic realities of virtual and hybrid work (Handke et al., 2024; Tanpipat et al., 2021). Development initiatives should integrate emotional presence, relational capability, and systemic care as core leadership competencies, equal in importance to strategic or operational performance. Metrics for leadership success should also reflect this shift, incorporating indicators of connection, engagement, and well-being as well as relevant performance outcomes.

In summary, sustaining leadership in virtual and hybrid environments is not the responsibility of individuals alone but a collective endeavour. Organisations that invest in the emotional and structural conditions for connection, and that enable leaders and teams to share the work of sustaining it, are more likely to achieve both lasting performance and authentic human cohesion in the evolving world of work (Castellano et al., 2021; Lin et al., 2017).

### Limitations and future research

While this study provided valuable insights, it is important to acknowledge its limitations, which present opportunities for further research. First, it involved secondary analysis of a dataset originally collected for a broader study on virtual and hybrid team performance (Coulston et al., 2025a). While the current analysis brought a fresh interpretive lens focused on leadership, the use of existing data inevitably limited the potential to explore contextual nuances or emergent dynamics not captured in the original design.

Second, while the study included participants from both public and private sector organisations, the sample was weighted towards the private sector, which may limit the generalisability of the findings. Furthermore, the cross-sectional nature of the research provided only a snapshot of participants’ experiences at a single point in time. As such, it cannot capture how leadership dynamics, perceptions, or relational patterns may evolve over time or in response to organisational or personal change.

Third, there is a potential for social desirability bias or the influence of culturally acceptable behaviours, including the downplaying of negative experiences, given the presence of other team members in the focus groups (Teh et al., 2023), as well as possible sample bias. All participants were currently employed and willing to reflect on their leadership experiences, which may have excluded the perspectives of those who had disengaged, left their roles, or been most negatively affected by virtual and hybrid leadership challenges. These missing voices may have offered additional insights into the risks of unmet leadership needs, emotional depletion, or structural failures.

Building on these limitations, future research should further explore and test the relational and systemic insights highlighted in this study. Longitudinal qualitative designs would be particularly valuable in exploring how leadership aspirations, tensions, and relational dynamics evolve over time, especially through key transition points, such as shifts in team composition, or organisational restructuring. Such approaches could surface adaptive patterns, relational fractures, or resilience strategies that are obscured in single time-point studies. In-depth narrative or case-based methodologies might also reveal how meaning-making processes and emotional labour shift as leaders and teams navigate ongoing uncertainty.

Mixed-methods studies may offer a valuable extension to these insights. While qualitative data remains essential for capturing the nuance and complexity of leadership as a lived, emotional experience, quantitative measures, such as burnout levels, psychological safety, or perceived boundary violations, could help empirically test the gain/loss cycles and resource dynamics suggested in this and related studies (Bakker et al., 2023; Mazzetti & Schaufeli, 2022). Furthermore, an intersectional lens is needed to examine how gender, race, cultural background, and professional role expectations shape the experience and burden of relational leadership. For instance, are some leaders more expected, or more penalised, for demonstrating emotional availability or setting boundaries. Such questions remain underexplored in the context of hybrid work and warrant greater attention to equity, inclusion, and labour care (Gabriel et al., 2023).

At an organisational level, future research could assess the impact of structural interventions aimed at sustaining leadership. These might include peer learning and coaching groups, leader well-being charters, cultural norms around feedback and flexibility, or system-level efforts to align workload with emotional and relational demands. Exploring the outcomes of such interventions, both for leaders and their teams, could help build an evidence base for more sustainable, human-centred approaches to leadership in virtual and hybrid environments (Grobelny, 2023). Finally, further conceptual development is needed to reframe leadership not just as an individual trait or role, but as an emergent, co-constructed process shaped by systemic conditions. Theoretical models that centre emotional presence, relational reciprocity, and structural support can help evolve the discourse on virtual and hybrid leadership towards collective sustainability and resilience.

## Conclusion

This study contributes to a growing body of scholarship that seeks to reframe leadership in virtual and hybrid contexts. Centring the lived experiences of team members, it positions leadership as a co-constructed, emotionally charged practice shaped as much by systemic conditions as by individual behaviours. The findings reveal a shared aspiration for emotionally present, responsive leadership; yet this ideal often remains precarious, with its weight unequally carried by leaders. When organisational systems fail to support the emotional and relational demands of leadership, the result is not only individual strain but also weakened trust, connection, and team cohesion.

Our insights challenge traditional paradigms that locate leadership primarily in output, authority, or technical competence. Instead, they highlight leadership as a relational practice shaped by systemic factors and sustained collectively. Leadership in dispersed settings cannot depend on individual effort alone; it requires shared responsibility, cultural permission, and structural alignment. If organisations aspire to leadership that is emotionally intelligent, available, and authentically human, they must create the conditions for leaders to show up fully, not as heroic figures, but as supported, seen, and co-responsible actors within a connected system. In doing so, leadership becomes not only a source of direction and cohesion but also a shared resource for connection, collective well-being, and leadership sustainability.

## Supplementary Material

Final Indicative question guide for focus groups.docx Final Indicative question guide for focus groups.docx

Final Indicative question guide for team leader.docx Final Indicative question guide for team leader.docx

## Data Availability

Anonymised data of all transcriptions are available from the corresponding author upon reasonable request.
